# Variability of response to the fluid bolus is again demonstrated

**DOI:** 10.1186/s13054-017-1793-z

**Published:** 2017-08-25

**Authors:** Ellis R. Muggleton

**Affiliations:** Rotkreuzklinikum München, Nymphenburgerstrasse 63, 80634 München, Germany

The AFRIM study by Obonyo et al. [1] presents haemodynamic data worthy of closer analysis. The inferior vena cava collapsability index (IVCCI) was used as a surrogate measure of intravascular volume status. However, this measurement is of limited use in spontaneously breathing patients, as demonstrated by a recent meta-analysis [2]. According to Table 1 from [1], 100% of group 1 and group 2 demonstrated chest indrawing leading to the generation of significant negative intrathoracic pressures and making the measurements invalid.

The purpose of a fluid bolus is to increase stroke volume and hence cardiac output. Although these patients were dehydrated, only 5 out of 11 in group 1 and 5 out of 9 in group 2 demonstrated a 10% increase in stroke volume following the fluid boli. Two patients in group 1 demonstrated a 10% *decrease* in stroke volume index, with a decrease of 48% in one case! It would interest us what the authors believe the reason for this effect is.

We believe that the administration of fluid, even in hypovolaemic patients, is more complex than often appreciated, with an interaction of many factors related to vascular biology, and further investigation is required to enable an optimisation of fluid delivery and to avoid the detrimental effects of fluid overload.

## Abbreviations

FR: Fluid responsiveness
IVCCI: Inferior vena cava collapsability index
