# Supplementary material for: Variability of response to the fluid bolus is again demonstrated
Source: Crit Care. 2017 Aug 25;21:224. doi: 10.1186/s13054-017-1793-z (PMC5574114; doi:10.1186/s13054-017-1793-z)
Supplement: Related texts

# Reply: Authors’ response

**Authors:** The AFRIM study authors

We are encouraged by the interest in fluid resuscitation in children with severe acute malnutrition and hypovolaemic shock, and have read with keen interest the contribution by Dr. Ellis Muggleton highlighting the limited utility of IVCCI in spontaneously breathing patients.

In the manuscript, while presenting these data [1], the authors noted the limitations in interpretation of the IVCCI, especially with respiratory distress characterised by deep (Kussmaul’s) breathing and chest indrawing. Given the limited ability of IVCCI to predict fluid responsiveness (FR), it is important to underline the fact that a single negative test with recognised limited utility cannot be used to rule out FR [2] and more research on non-invasive assessment of FR is needed as there is still emerging research advocating utility of IVCCI [3].

Reduction in stroke volume index following fluid bolus administration is indeed an unexpected finding. Nonetheless, our findings stimulate a new direction of inquiry investigating the mechanisms underlying the pathophysiology of hypovolaemia with severe malnutrition as well as the interpretation of the patients’ relative position on the Frank-Starling curve with respect to FR. This is particularly important in the context of little understanding on the effect that fluid administration has on the complex patho-biological interaction of intravascular hypovolaemia and severe malnutrition. Previous research has shown that hypovolaemia and dehydration are associated with higher mortality in severe malnutrition [4]. The AFRIM study showed no supportive evidence of increased risk of cardiac failure in severely malnourished children receiving fluid resuscitation for hypovolaemic shock [1]. The patient with a stroke volume reduction of 48% also had an increase in the systemic vascular resistance index of 56%, which could be indicative of an advanced stage of illness with extreme physiological compensation.
